# The Influence of Signals on Donation Crowdfunding Campaign Success during COVID-19 Crisis

**DOI:** 10.3390/ijerph18147715

**Published:** 2021-07-20

**Authors:** Han-Chiang Ho, Candy Lim Chiu, Somkiat Mansumitrchai, Zhengqing Yuan, Nan Zhao, Jiajie Zou

**Affiliations:** College of Business and Public Management (CBPM), Wenzhou-Kean University, Wenzhou 325060, China; hho@kean.edu (H.-C.H.); smansumi@kean.edu (S.M.); yuanzhe@kean.edu (Z.Y.); zhaona@kean.edu (N.Z.); zouji@kean.edu (J.Z.)

**Keywords:** crowdfunding, donation-based campaign, COVID-19, Coronavirus, food relief, signaling theory

## Abstract

In 2020, the coronavirus pandemic devasted public health agencies and the federal government across the world. Bridging the gap between underserved populations and the healthcare system, the donation-based crowdfunding campaign has opened a new way for suffering individuals and families to access broader social network platforms for financial and non-financial assistance. Despite the growing popularity of crowdfunding during the pandemic crisis, little research has explored the various signals that attract potential donors to donate. This study explores the effects of signaling theory on the success of a crowdfunding campaign for food relief launched in GoFundMe during which the United States was severely affected by the pandemic with a surged number of coronavirus infected cases from 1 March with 134 confirmed COVID-19 infected cases to 29 July with 4,295,308 infected cases according to World Health Organization. The following results show that the three different signal success measures are important to the success of crowdfunding campaigns: (1) signals originating from the campaign (Title, Description, Spelling Error, Location, and Picture); (2) signals originating from the fundraiser (Social Network, and Update); and (3) signals originating from the social interaction of the fundraiser with the crowd (Comment, Follower, and Share). These findings provide insight and bring additional knowledge contribution to the crowdfunding literature.

## 1. Introduction

During a time of disaster or tragedy, victims and survivors who do not have adequate resources to support themselves and their families often rely on their networks, as government and charitable support may be slow or even lacking. The crowdfunding platforms allowed anonymous internet users to provide help and donation to the fundraisers directly. Organizing financial assistance online through crowdfunding platforms (e.g., GoFundMe, Kiva, YouCaring) has emerged as an alternative and effective method to seek support and has become a popular form of caregiving. The current largest social fundraising platform, GoFundMe.com, raised over USD 9 billion by individuals since its inception in 2010 (see http://www.gofundme.com/, accessed on 3 March 2020). In the United States, donation-based crowdfunding (DbC) accounts for USD 293.5 million of financing [1]. Berliner and Kenworthy [2] stated the rise of medical or DbC in the United States for two reasons: the first is the fiscal crisis in American health care during the 2008 global financial crisis. In addition, the second, the growing social assistance systems of online social networks.

Based on Pew Research Center’s study [3], one out of five Americans has donated to a crowdfunding fundraising campaign. One of the most well-known and most extensive DbC campaigns is the project to support the individuals affected by Hurricane Harvey in the United States [4]. As stated by Conway [5], when National Football League (NFL) star Justin J. Wyatt raised over USD 37 million from over 200,000 donors who donated to aid those affected by Hurricane Harvey in 2017. He accomplished this feat by organizing a Houston Flood Relief Fund crowdfunding page at the YouCaring crowdfunding website with a target goal of USD 200,000. Even after the deadline, donations continued to flow, reaching a total of USD 41.6 million [6].

The phenomenal success of crowdfunding emerges in the context relative to the funding of goods, resources, services, or to address social problems primarily related to monetary resources’ contribution through collective efforts in the digital setting [7]. As stated by Johnson and Puplampu [8], the Internet as a form of technology is a part of an ecological techno-subsystem where individuals interact with both non-human (i.e., crowdfunding website) and human (i.e., donors) sources of information which allows continuous reciprocal interaction. Concerning DbC, campaigns (e.g., arising from accidents, crises, disabilities, memorials, and illnesses) are initiated by family, relatives, or friends on the recipient’s behalf to support a humanitarian cause [2]. The reward of DbC for donors is more of an emotional or self-actualization nature such as feeling good, a sense of satisfaction, alleviation from guilt, pro-social, warm glow, or public acknowledgment about helping others in need in times of difficulty [9,10]. It is complicated for potential donors to evaluate the DbC campaigns’ quality over non-charitable campaigns because it is a voluntary act where donors do not expect any monetary or tangible return benefits or pay-off [11].

With the recent impact of the COVID-19 far beyond those infected by the virus, DbC platforms have become a powerful tool or alternative source for various personal and public-related causes to raise funds and donate funds due to their extensive reach. Based on the study of Rajwa et al. [12] of 1579 COVID-19 campaigns on GoFundMe.com from the 3rd of March to the 20th of March 2020, the total fundraising amount was almost USD 1.5 billion, and more fundraisers are actively organizing campaigns with the spread of the virus. They also mentioned that 88% of those campaigns focus on losing a job, unable to cope with living expenses and food relief. For this reason, crowdfunding is a crucial survival strategy for needy individuals to market their basic needs during a natural disaster. However, potential donors can view thousands of COVID-19 campaigns on different DbC platforms, which entails information overload. For potential donors to effectively sort through thousands of DbC campaigns, fundraisers need to avoid information overload, complicating the assessment of potential donors to discern the truly in need fundraisers, so sometimes they rely more on given signals.

Earlier studies on crowdfunding focused on signaling theories because crowdfunding projects are characterized by high information asymmetry [13,14,15,16]. Given different donation campaigns characterized by unobservable attributes, which observable signals lead to donations in specific campaigns and not others? Some are very successful, while others are not. As Kunz et al. [17] stated, crowdfunding campaigns use a trial-and-error approach to analyze the factors influencing the project’s success and failure. Therefore, there is a need for an in-depth analysis of these factors for the success of donation-based campaigns. In addition, there is little research on the signaling strategies of DbC campaigns during a natural disaster. Most previous studies about DbC campaigns are done before the COVID-19 pandemic [9,10,18,19,20,21].

Therefore, motivated by this knowledge gap, this study explores the DbC campaign signals and attributes that donors evaluate from massive information provided by the fundraisers, which induce them to commit financial donations. To successfully raise funds via a DbC platform, fundraisers will need to find ways to signal their cause to the mass public and capture the attention of potential donors. This knowledge gap is filled in this research by linking signaling theory and DbC during the pandemic crisis. This study provides supporting evidence of the relative importance of different types of antecedents of success in the DbC context. Thus, this study not only focuses on the effects of the drivers per se but also on the effects of specific characteristics, such as the number of words per title, the number of words of the campaign’s description, and the location where relief will be donated. Knowing which drivers influence the design of an effective campaign and donors’ participation is crucial for relief efforts to be successful. Especially in the wake of the global pandemic crisis, understanding self-marketing or self-promotion for survival requires DbC fundraisers to learn the different success factors to attract mass public support.

To achieve this purpose, this article is organized as follows: first, it provides a comprehensive overview of the theoretical framework concerning the interrelationship between success factors and the probability of success. Based on the literature review, we classified set quantifiable signals (i.e., signals originating from the campaign, signals originating from the fundraiser, and signals originating from the social interaction of fundraiser with the crowd) based on previous research on crowdfunding and formulated hypotheses regarding the effect of the signals on the campaign’s success. Second, the empirical data were derived from crowdfunding campaigns, particularly food relief projects launched in GoFundMe.com between 1 March 2020 to 29 July 2020, during which the United States was severely affected by the COVID-19 pandemic with an exponentially surged number of COVID-19 infected cases from March 1st (i.e., 134 confirmed COVID-19 infected cases) to July 29th (i.e., 4,295,308) [22]. Third, the data were analyzed using logistic regression. Lastly, we conclude this research by providing theoretical and managerial implications and identifying future research directions.

## 2. Literature Review

### 2.1. Success of the Campaign

In the context of crowdfunding campaigns, success is a measure when the amount requested reaches its funding goal within the target period [23]. Crowdfunding platforms can choose either the “all-or-nothing” model or the “keep-it-all” model to run the campaign. In the “all-or-nothing” model, the project owner receives the invested money only if the minimum necessary fund is achieved or greater than the target amount [24]. The project owner can keep any funds that the project collected under the “keep-it-all” model [25]. For example, in GoFundMe as a donation-based campaign, reaching the campaign’s goal is not required. The fundraiser or charity can keep every donation they receive, whether a favorable outcome or not. The donation campaign can still accept donations even after the goal is reached. In addition, even though the fundraiser would like to continue raising money, they can still keep running the campaign for as long as they like. If the fundraiser wants to increase the funding goal, the fundraiser can increase and post an update on the campaign website explaining why they made the changes and how the additional donations will be used.

Achieving the success of a campaign is an indicator of crowdfunding performance [23]. In this paper, the campaign succeeds if the donation amount is greater than or equal to 100%. Thus, if the fundraiser receives at least the initial set of target amounts, the campaign is successful. The difference of DbC from other forms of crowdfunding (e.g., equity, reward, and length) is that the contributors or donors usually receive no benefit other than the feeling of satisfaction from doing good deeds for others. Therefore, fundraisers need to put more effort and execute more marketing strategies to induce crowds to donate so they can achieve their target goals [9,26]. Overall, this study will identify various project quality signals of a donation-based crowdfunding campaign and their influence on project success.

### 2.2. Signaling Theory

Based on the study of Ross [27] and Spence [28] on signaling theory, one party will determine how to communicate and signal the information while the other party will decide how to interpret this sort of information. Connelly et al. [29] further pointed out that the signaling theory can reduce information asymmetry’s negative effect when a better-informed party communicates and transmits a high-quality signal to the less-informed party. Crowdfunding signals present quality of content that influence crowdfunding project success [30]. Fundraisers are limited to a standardized structure of campaign presentation, limiting the amount of information shared with potential donors, which gives rise to information asymmetry and uncertainties [15,31]. Uncertainties are very significant and prevalent among crowdfunding projects, especially when donors want clear information on how their donation efforts will be used for the project [32,33].

For relief donations, information asymmetry concerns are critical because the donors can be informationally disadvantaged regarding the fundraiser’s credibility and behavioral tendency to use the donated money as promised. Some crowdfunding campaigns are used to prey and take advantage of donors’ kindness through fraudulent campaigns. For example, the case of the GoFundMe scam caused by a South Jersey couple (Katelyn McClure and Mark D’Amico) who raised $400K for a homeless veteran (Johnny Bobbitt) from a fabricated DbC campaign titled “Paying it Forward” to further the scheme, they did numerous local and national media publicities to attract potential donors [34].

Funders rely on quality signals [35]. In order to mitigate the challenges of information asymmetry and uncertainties, the fundraisers deliberately send quality signals and disclose information about unobservable attributes of the projects to guide potential donors in decision-making [14,16,36]. Various signals in DbC campaigns force potentials donors to identify worthy causes for donation [21]. Signals should be highly visible, observable, process ongoing, and frequently communicated [14], which are highly dependent on the effectiveness of the message conveyed from the fundraiser to anonymous donors regarding whether the information accessed by both parties is consistent with the campaign initiatives. There are identifiable project quality signals that can predict campaign success.

### 2.3. Signals Originating from the Campaign

As stated by Defazio et al. [37], in a digital environment like a crowdfunding project, “each project appears as one piece of a mosaic displayed by its title, photo and blurb—a short promotional phrase—with a hyperlink that directs to the project’s page.” The project has a specific fundamental part that builds the framing of information in a hierarchical structure. The donors see the whole project content and hierarchically process the information and then decide whether to support it or not. Therefore, fundraisers need to invest more significant efforts to describe the project’s quality through specific attributes associated with the project itself and give more attention to various fundamental parts of the campaign to achieve success [38]. Fundraisers can communicate static information and also establish a visual narrative as prescribed by the specific requirements of crowdfunding campaign-related features.

#### 2.3.1. Title of the Campaign

The significance of the textual title or heading of a crowdfunding campaign that the potential donors view first will influence their decisions about whether to continue browsing the rest of the campaign information later or immediately answer the fundraiser rallying cry. Concerning how magazine advertising works, the images created by the title words and phrases associate readers with the topic that creates its unique positioning [39]. The similarity of a short promotional phrase campaign to the importance of the 140-character limit of Twitter posts is comparable to browsing newspaper headlines. Compressing the details of campaign titles and maximizing the limited spaces make every word and every character count. Readers of Twitter posts passively digest tidbits of factual information than actively processing them [40]. Defazio et al. [37] stated that crowdfunding campaign title is crucial to get the crowd’s attention and reading comprehension, helping contextualize their expectations on the narrative description content. Based on the tips of GoFundMe [41] on the fundraiser title, it should be short with four to eight words, descriptive, and inspiring for people to find it easy to search online. Therefore, based on previous studies, we assumed that the length of the title based on a number of characters could influence the campaign’s success. Therefore, we hypothesize:

**Hypothesis** **1** **(H1).** *The crowdfunding campaign with more characters on the title will positively cause a higher probability of success*.

#### 2.3.2. Project Length of Textual Description

The textual description of the project is an essential narrative of a shared campaign with the crowd or potential donors. Previous studies found that the longer word count of descriptions in crowdfunding campaigns can deliver more information and have a significant positive influence on project success [17,42,43,44]. It also reduces information asymmetries and increases funding probability by 5% [45]. According to Lagazio and Querci [46], using greater than 500 words in the project description increases the likelihood of success by 13%. Based on the prescribed description length of donation projects [47], at least 400 words is the minimum length to keep the crowd reading. The more contents (i.e., 800 words better, and around 1000 words is ideal) the fundraisers write on the donation page, the easier people find in an online search such as Google. A short description of a fundraising campaign provides insufficient information for the crowd to evaluate the campaign and look less trustworthy. Based on the findings of Barbi and Bigelli [48], the results showed that funded projects have an extensive description of 7109 characters, including spaces, while unfunded projects have 6295 characters. Therefore, they concluded that the fundraiser spent significant time and effort in explaining the campaign to the crowds, signaling the project’s quality. If the project description word count exceeds 2013, the likelihood of campaign success becomes negative [43]. As longer information is provided, it may increase the complexity of understanding the vision of the fundraiser. However, they also found out that project descriptions are too extensive that can signal the fundraiser’s lack of conciseness because it becomes detrimental to attracting donors or backers. Therefore, we hypothesized that:

**Hypothesis** **2** **(H2).** *The crowdfunding campaign with more word count in the description section will positively cause a higher probability of success*.

#### 2.3.3. Presence of Any Spelling Error

The existence of spelling errors signals the low quality of the crowdfunding campaign, the fund seeker’s unpreparedness, and less committed fundraisers [15,43,49]. Concerning peer-to-peer loan requests, spelling errors appear that the project’s applicant may even appear to be untrustworthy [45]. In addition, spelling errors on the website and document signals a low-quality project [50]. Drawing from a large dataset of Kickstarter projects, the existence of spelling errors can also decrease the chances of success by 13% [23]. Sulaeman and Lin [51] showed that fewer spelling errors on charitable fundraising campaigns lead to more donors contributing and a higher total amount of donations. According to GoFundMe [47], since most of the crowd uses online searches for keywords to find a cause to support, spelling is a critical element for Google’s algorithm screening process to precisely detect your campaign to give the most relevant answers to the people query. Therefore, a donation campaign with the presence of spelling errors within the description text is less likely to be funded. Based on these, we propose:

**Hypothesis** **3** **(H3).** *The presence of any spelling error will negatively affect the successful probability of a crowdfunding campaign*.

#### 2.3.4. Geographic Location

In an emergency or disaster situation like the COVID-19 pandemic, food shortages, supply disruption, and food price inflation exacerbated food disparities among the highly infected areas. Providing much-needed assistance to the highly infected areas or geographical constraints has always been prioritized. Fundraisers and donors in crowdfunding platforms are geographically dispersed people looking forward to receiving funding for a project and willing to support a project. Mollick [23] stated that crowdfunding campaigns are not evenly distributed but more concentrated. The highest donation value comes from the exact donor geographic location, nearby areas, or donors with personal ties geographically closer to the fundraisers [52]. The geographic preference of donors such as “home-bias” where lenders tend to do transactions to borrowers who are geographically closer because it is easier for lenders to evaluate and build trust due to similar backgrounds [53,54]. The geographic location of a campaign is only associated with the amount pledged per donor in minor instances, and it still depends on the nature of the crowdfunding [55]. Guo et al. [54] studied distance diffusion of home (e.g., country, state, or city) bias from different types of crowdfunding projects; 90% of donors only invest in food projects from their own country. Therefore, motivated by the rise in donation crowdfunding during the COVID-19 pandemic and the broad geographic dispersion of the COVID-19 infected areas, this study will explore the variable geographic location because donation campaigns launched from highly infected areas versus those from low-infected areas, the former may tend to attract a higher number of donors or amount of donations. Hence, we hypothesize:

**Hypothesis** **4** **(H4).** *The region with more COVID-19 infected numbers will receive more crowdfunding donations*.

#### 2.3.5. Visual Presentation of Picture and Video

A visual presentation is an addition to the voluminous amount of information from the textual description of the project campaign, which contributes to the understandability of the project’s goal and providing a sense of reality [17]. A textual description offers detailed explanations of the project, whereas a visual presentation concisely outlines a crowdfunding campaign [46]. Visual images are essentials for a pitch competition and crowdfunding context [43,49]. Crowdfunding platforms encourage fundraisers to initiate visual presentations like pictures, video-recorded pitches, infographics, and animation [42]. These elements stimulate various sensory channels that are appealing to the crowd [32] and enhance media richness (e.g., images, designs, or colors) in online communication [44]. Donors interpret a fundraising campaign with a video presentation as an attribute of project quality [56]. A visual presentation is an indicator of credibility and preparedness to live up to the promise [15]. A fundraiser or an organizing team who put time, effort, and designing into preparing a visual pitch are more likely to succeed [57]. Mollick [23] shows that campaigns without a video included decreasing the probability of success by 26%. As stated by Berliner and Kensworthy [2], the success of a donation campaign based on the suggestion of GoFundMe is to use self-produced visual content around a specific problem that a donor can feel sentiment that they are helping to solve. Based on these, we propose:

**Hypothesis** **5** **(H5).** *The presence of any picture will positively affect the successful probability of a crowdfunding campaign*.

**Hypothesis** **6** **(H6).** *The presence of any video will positively affect the successful probability of a crowdfunding campaign*.

### 2.4. Signals Originating from the Fundraiser

When the DbC is limited to the crowdfunding homepage display, the emergence of the fundraiser’s external websites and social networks can reduce information asymmetry [46]. As stated by Davies and Giovannetti [16], potential donors are unable to clearly observe the fundraiser’s background or activities beyond their self-representation through their campaign website on the crowdfunding platform. Hence, by signaling about their background, positive intention to help, and past campaigns through an external link or social media, fundraisers may reduce information asymmetry. Potential donors can also draw meaningful inferences about the current DbC quality because they can access more detailed information that shows an effort by the fundraiser to signal more transparency [26,46].

#### 2.4.1. Presence of Any Social Network

Social networking is at the center of a donation-based crowdfunding platform, such as GoFundMe, a marketing tool for fundraisers [2]. Most crowdfunding campaigns leverage multi-platform social network sites such as Instagram, Twitter, Pinterest, LinkedIn, Snapchat, Facebook, and YouTube links that encourage users to share and circulate the information to the public [58]. Crowdfunding platforms also encourage fundraisers to share their campaigns through social media to increase visibility to the mass public. Kromidha and Robson [55] stated that when a fundraiser presents their social identity within a social network, it shows their aspirations to place them in a favorable position that would secure more significant amounts of pledges from the donors. Donors can search and source more detailed information that can play an essential role in funding decision-making [15]. Social media networks embedded in the campaign can have higher online searchability. Fundraisers provide campaign updates that generate electronic word-of-mouth (e-WOM) to spread information virally [23]. Donors can also use social media to screen promising projects by checking the number of the fundraiser’s friends [59]. The number of social media friends shows the fundraiser’s potential to mobilize collective actions from the crowd, enhancing benevolence and trustworthiness [17]. The study of Lagazio and Querci [46] demonstrates that the probability of success may reach 43% if the number of Facebook shares ranges from 1000 to 10,000. Furthermore, if it is more than 10,000 shares, the effect may reach nearly 80%. Based on the previous results and implications, we propose the hypotheses for this study are:

**Hypothesis** **7** **(H7).** *The presence of any social network link will positively affect the successful probability of a crowdfunding campaign*.

#### 2.4.2. Presence of Any External Website

An additional external website (e.g., personal homepage) for fundraisers can provide more relevant information, such as narrating their detailed stories or hardships to encourage the crowds to donate. Fund seekers must give all needed information (e.g., an external website link) to help in the decision-making of potential donors to mitigate information asymmetry [32,44,46]. An external website signals transparency because it provides more detailed and additional information about the project and the fundraisers that can generate traffic to the fundraiser’s homepage, enhance communication with stakeholders [26], and increase trust. After all, it serves as the creator’s characteristic [36]. Potential donors are usually restricted to the information available from the crowdfunding platform. Links to external websites can enhance the functionalities of communicating with the public [17] and increase the probability of success by 6% [46]. Fundraisers can customize the design for project presentations, especially for target donors. Therefore, having a link to an external website may increase the probability of project success. Thus, we propose a hypothesis:

**Hypothesis** **8** **(H8).** *The presence of any external website link will positively affect the successful probability of a crowdfunding campaign*.

#### 2.4.3. Presence of Any Update

Fundraisers communicate with their supporters to provide all necessary information by posting updates of their projects that everyone can see. It usually appears on a separate section where fundraisers provide personalized messages such as saying “thank you” to donor/s, presenting the campaigns’ progress or status, and sharing communication materials (e.g., blogs, photos, pitch videos, social network links, events to participate). The update is a one-sided communication tool that enables fundraisers to deliver their campaign’s value and legitimacy, influencing the campaign’s trustworthiness and credibility [14]. The presence of any update may increase the probability of success by 5% [46], while the absence of an update reduces the chance of success by 13% [23]. The frequent information updates of the fundraiser regarding the status of the project are associated with the campaigns’ successful performance [60]. In addition, the value of updates to potential donors will decrease as the fundraiser no longer provides new updates [14]. As Kunz et al. [17] stated, the reward crowdfunding campaign initiator should keep sending project updates during the pre-funding phase no matter whether the campaign is running or close to the goal. Even during the post-funding phase, it is critical to put the donors in the loop as a sign of signaling effectiveness. Hence, we hypothesized:

**Hypothesis** **9** **(H9).** *The presence of any update will positively affect the successful probability of a crowdfunding campaign*.

### 2.5. Signals Originating from the Social Interaction of Fundraiser with the Crowd

According to Clauss et al. [60], the campaign’s success depends on convincing the anonymous public through dynamic social interaction. Social interactions such as shares and likes on social networks serve as supplemental information such as the textual campaign description, visual presentation, and general information, which are adequately critical to support the project’s success. In addition, fundraisers’ social ties signify quality signals and are a good predictor of successful project backing [23]. As stated by Belleflamme et al. [35], a more extensive network of connections is significantly connected with unobservable project quality components; therefore, it serves as a signal. Several scholars [15,24,30,36,60,61] found that fundraisers who provide greater awareness through social interactions are considered an attribute of the effectiveness of the signal and predictors of campaign success.

#### 2.5.1. Presence of Any Comment

The crowdfunding platform comment section performs a dialog or joint conversation forum between backers or donors and fundraisers in which fundraisers can provide a direct response to the crowd’s feedback. Dikaputra et al. [26] defined “comment” as electronic word-of-mouth. The backers, donors, or the crowd can ask questions and seek clarification, while the fundraisers can express appreciation, answer questions, and post an explanation. Based on the study of Clauss et al. [60], posting comments, whether positive or negative, can facilitate dynamic interaction between parties, and it can communicate the strength of the crowdfunding campaign because the crowd can share their feelings, perceptions, and others feedback to create a shared understanding of the campaign. Anonymous crowd comments can also impact a potential donor’s funding decision and generates support [62]. Courtney et al. [15] prove that the donors’ sentiments reflected in their comments help mitigate information asymmetry. The on-site communication (i.e., comment section) helps the crowd understand more about the campaign and gain more confidence [58]. An engaging joint forum between the fundraiser and its donors could achieve a more excellent pledge/backer ratio [49]. According to Wang et al. [63], fundraiser’s comment quantity, reply to length and speed, as well as comment score, are significant to the project’s success. For donation-based campaigns, donors’ comments reinforce the worthiness of the cause [2]. Therefore, we assume that posting comments will have a signaling effect on the crowd and increase the probability of a crowdfunding relief campaign’s success. Hence, we hypothesize:

**Hypothesis** **10** **(H10).** *The presence of any comment will positively affect the successful probability of a crowdfunding campaign*.

#### 2.5.2. Followers and Shares

A popular campaign on crowdfunding sites with a number of followers and shares can attract potential backers or donors [44]. The follower and share buttons on the crowdfunding platform page placed on each campaign’s dashboard are examples of a crowd engagement enabler on an online platform [64]. It is significant to measure how potential and active donors convert emotion and cognition into actual action, making donors’ emotions visible and shared [65]. The goal of these marketing functions is the following: (1) for random visitors to share the project online through social media or email; (2) for potential donors to share online; (3) for existing donors to share online; and (4) for committed donors to share online. Crowd endorsements and social influence can complement and verify campaign information, decreasing information asymmetries [56]. Following and sharing information means more online promotions spread the project; it shows how social influence and e-WOM spread effectively to the anonymous public [17,23]. An organic process of clustering similar interests and recruiting others is a form of social identity theory that influences participation, relationship, interaction, and commitment [55]. Xie et al. [66] expressed that the number of crowdfunding campaign followers positively affects funding amount. Therefore, we hypothesize as follows:

**Hypothesis** **11** **(H11).** *The crowdfunding campaign with more followers will cause a higher probability of success*.

**Hypothesis** **12** **(H12).** *The crowdfunding campaign with more shares by viewers will cause a higher probability of success*.

**Figure 1:**
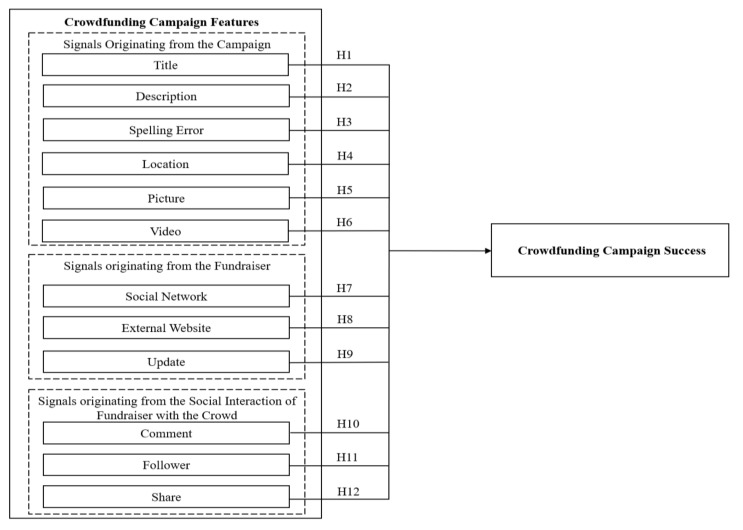
Proposed research model.

Based on the literature and theoretical framework discussed above, this study proposes a conceptual framework shown in Figure 1.

## 3. Materials and Methods

### 3.1. Data

We used a web data extraction software like Python programming to collect data, similar to previous crowdfunding studies [63] from GoFundMe, one of the world’s leading DbC platforms. Web data extraction software helps collect, filter, and clean large secondary datasets based on the given keywords with high validity and provides quality reports for data analysis and visualization. The data focused on the COVID-19 food relief campaigns in the United States from 1 March 2020, to 29 July 2020, during which the United States was severely affected by the COVID-19 pandemic with an exponentially surged number of confirmed cases.

Although there are different active crowdfunding platforms for fundraising food relief campaigns in the world, such as Kickstarter, Patreon, GoFundMe, Indiegogo, and so on, we select to focus on the coronavirus food relief campaigns from GoFundMe in the United States based sample for some main reasons: First, unlike other crowdfunding campaigns, food relief campaigns started several years ago; the idea of food donation is easy to be recognized, and highly possible to make statistically significant inferences. Second, the GoFundMe platform is one of the most trusted and powerful online fundraising platforms. Since 2010, it has started with a crowdfunding campaign on the site with over $10 billion raised and designed to help small and large groups raise money together. The platform also verifies the authenticity of the need. Therefore, the data from GoFundMe is reliable. Third, the United States society is a typical big open society. With the advanced development of Internet technology, information delivery is public and transparent. Consequently, donors and receivers could understand and follow the crowdfunding process easily. Fourth, web data extraction has a high validity to collect large datasets because the website (i.e., GoFundMe) is always working properly, and the data are generated by web-based transmission [63]. Fifth, since the outbreak of COVID-19, many people have lost their income and do not have food to fill their families. Food donation could provide some relief for struggling and overlooked people. It is a meaningful activity to help people who genuinely need it. Sixth, in terms of possible future research, the USA data are persuasive and generalized. Those situations make GoFundMe and the USA data particularly appropriate for the analyses and give a clear route for future academic research about crowdfunding research.

For collecting valid data, this research set two main criteria. First, the crowdfunding campaign content should be related to food relief campaigns under COVID-19 subjects. Second, the money donation should go to people who live in the USA. The initial samples contained 4945 ongoing food relief campaigns on GoFundMe. After double-checking all samples derived by Python for data cleaning (e.g., money could only help people in the United States) and manually identifying the study’s data availability and requirement, the available sample size included 686 campaigns. In addition, the extreme amount of money values of fundraising targets was deleted because those campaigns appeared non-thoughtful to raise funds (e.g., five campaigns with targets less than $100, and twenty-three campaigns with targets over $500,000, none of which came closer than 0.3% of their targets) [67]. The final sample included 652 campaigns, of which 29.9% were made into successful campaigns. The remaining 70.1% of the campaigns were returned to the backers (i.e., all-or-nothing model) because campaigns did not achieve the minimum target [23]. With the help of the Internet overcomes geographical barriers and allows fundraisers to reach a significant number of potential backers to participate in crowdfunding campaigns worldwide. The total fundraising goal from GoFundMe for the COVID-19 food relief campaigns was $12,975,214 alongside the $6,348,256 total donation received from 62,495 donors, 158,952 shares, and 63,587 followers as a whole from the 652 collected crowdfunding campaigns. Seeing the sample data more specifically, the median of the fundraising goal is $6658, the median donation received is $1925, while the median number of donors is 36. Regarding the nature of the COVID-19 food relief campaign, the majority of the collected samples are for buying food (87.8%), buying and making food (3.2%), and making food (9.0%).

Based on the criterion of the U.S. Census Bureau [68], the USA continent is divided into four main regions, the Northeast region (e.g., New York State and Pennsylvania State), West region (e.g., California State and Colorado State), Midwest region (e.g., Ohio State and Kansas State), and South region (e.g., Texas State and Florida State). In terms of the number of COVID-19 infected cases, during the data collection period, the website of the Centers for Disease Control and Prevention [69] of the USA showed that there were 215,532 infected cases in the West region, 367,675 cases in the Midwest region, 446,108 cases in the South regions, and 564,235 cases in the Northeast region. Table 1 demonstrated the sample characteristics collected from the GoFundMe platform.

### 3.2. Variables

#### 3.2.1. Dependent Variable

The main target of a crowdfunding campaign is to get money to help people. Hence, a successful crowdfunding campaign aims to attract a sufficient amount of raising [23]. It is one of the most popular measures for the success of a crowdfunding campaign [15]. As a result of this, measuring campaign success is whether the campaign can raise equal to or higher than the minimum target (i.e., the amount received at the end of the crowdfunding campaign). Based on the literature, the dependent variable is the success of a crowdfunding campaign and was coded as 1 = success and 0 = failure.

#### 3.2.2. Independent Variable

The target is to exam the interaction effects between fundraisers and backers on the successes of crowdfunding campaigns. The selection of variables to explain the successes of crowdfunding campaigns is mainly based on previous research suggested variables and the available functions provided by GoFundMe to make sure that some critical factors have not been missed. Here, the variables are the arbitrary information provided by fundraisers to backers to generate the interaction effects. Furthermore, the variables are included in this research, which could eliminate the possible effects of some antecedents to influence the performance of crowdfunding campaigns [36]. To better explain the relationships between independent variables and the success of a crowdfunding campaign. In this study, three kinds of variables are proposed:

First, variables for signals originating from the campaign. The variable names are (1) Title, (2) Description, (3) Spelling Error (SE), (4) Location, (5) Picture, and (6) Video. Second, variables for signals originating from the fundraiser. The variable names are (1) Social Network (SN), (2) External Website (EW), and Update. Third, variables for signals originating from the social interaction of fundraiser with the crowd. These three variables are (1) Comment, (2) Follower, and (3) Share.

Those variables have been examined from previous studies and further applications in the coronavirus context to identify whether these factors are significant influencers to the successes of COVID-19 food relief campaigns. Table 2 reports the detailed descriptions of those variables.

In this study, successful crowdfunding campaigns represent about 29.9% of the sample (*n* = 652). Most crowdfunding campaigns show the presence of spelling errors (64.1%). The majority of the fundraisers supplement pictures (67.8%), videos (50.8%), social network (66.4%), external website (49.7%), comments (46.8%), and post updates (38.5%) to increase the campaign content and let potential backers understand more information about the campaign. Besides, the follower, share, title, and description are considered proxy variables.

The data of crowdfunding campaigns show that 251 to 500 followers (43.3%), 1001 to 1500 shares (38.5%), four to five title words (49.5%), and 601 to 900 words on the description (60.4%) are the significant percentages in the data sample. The crowdfunding campaigns would like to help people who live in the Northeast region (39.0%), South region (31.0%), Midwest region (17.2%), and West region (12.9%). The summary statistics are shown in Table 3.

Table 4 is a correlation matrix with all variables. The correlation analysis demonstrated the relationship between the independent variables and the dependent variable (the success of the crowdfunding campaign). It shows that while the presences of video and external website links have insignificant correlations with the success of the crowdfunding campaign, other variables showing significant correlations exist. The spelling errors have a significant and negative correlation with the success of the crowdfunding campaign, indicating that sloppy writing will cause an inverse effect on the campaign. The correlation coefficients are not high, far below 0.7, which implies no serious problems about multicollinearity [70]. All the variance inflation factor (VIF) values of the six estimated models with 1.7 were the highest individual VIF in any model, under the recommended cut-off value of 10 [71], confirming the issues with no multicollinearity as well.

### 3.3. Research Method

Logistic regression is an appropriate method for measuring hypotheses because the dependent variable is binary and independent variables consist of binary variables [72]. Logistic regression could identify what variables directly affect determining the proposed event (i.e., the coefficients of regressions are odds ratios) in order to examine the impacts of variables and build models that will predict these effects on a proposed event. This research uses the following logistic regression model:

The success of a crowdfunding campaign = β_0_ + β_1_ Title + β_2_ Description + β_3_ Spelling Error + β_4_ Location + β_5_ Picture + β_6_ Video + β_7_ Social Network + β_8_ External Website + β_9_ Update + β_10_ Comment + β_11_ Follower + β_12_ Share + ɛ, (1)

where the control variables are update, comment, follower, share, title, description, and regions. This study built six different logistic regression models. Model 1 includes all control variables other than independent variables with crowdfunding campaign success as the dependent variable. From Model 2 to Model 6, we add one more independent variable one by one to test the hypotheses, Model 2: Spelling Error, Model 3: Picture, Model 4: Video, Model 5: Social Network, and Model 6: External Website. Model 6 presents the full model.

## 4. Results

### Logistic Regression Results

According to the likelihood ratio chi-square test, the results show that the full model represents a significantly better model fit than the null models. Among the six models, the degrees of freedom of Chi-square tests are from 17 to 22, and Chi-square coefficients range from 53.761 to 65.598, *p* < 0.05, respectively. The six models explained range from 28.8% to 34.5% (Nagelkerke *R*^2^) of the variance in the success of crowdfunding campaigns. The significant improvement in model fit compared with the null models and significant *R*^2^ scores demonstrate that independent variables are an essential and significant portion of the dependent variable in each model (See Table 5).

All hypotheses were examined in the six models. Regarding title words, this study sets the dummy 1–3 title words as the reference group. Only the 4–5 title words (*p* < 0.05) are significantly shown 1.762–1.812 times high than 1–3 title words to increase the likelihood of the success of crowdfunding campaigns. The other two categories of title words, 6~7 title words (*p* > 0.10) and ≥ 8 title words (*p* > 0.10), are insignificant, which implies the effect of those two categories are similar to that of 1–3 words. H1 is partially supported.

The campaign description of 1–300 words is set to be the reference group. The campaign description of 601–900 words (*p* < 0.05) is 1.263–1.271 times higher than that of 1–300 words to increase the likelihood of the success of the crowdfunding campaign. However, the campaign description of ≥ 901 words (*p* < 0.10) is negatively significant. The inverted odds ratios for this dummy variable expressing that the odds of the description of 1–3 words are 1.001–1.038 times higher than that of ≥ 901 words. In addition, the campaign description of 301–600 words (*p* > 0.10) is insignificant, indicating that the effects of 1–3 words and 301–600 words on the success of crowdfunding campaigns are similar. Therefore, H2 is partially supported.

In Models 2 to 6, spelling error is a negative and significant (*p* < 0.05) predictor of the likelihood of the success of crowdfunding campaigns. The odds ratio indicates that for each one-unit increase on a spelling error, the odds of the success of crowdfunding campaigns increased by a factor of 0.656, 0.660, 0.660, 0.669, and 0.672, respectively. Hence H3 is supported.

About the region, it is set the Midwest region as the reference group. Money denoting to the Northeast region (*p* < 0.05) is 1.864–1.894 times higher than that to the Midwest region to increase the likelihood of crowdfunding campaign success. Furthermore, money denoting to the South region (*p* < 0.05) is 1.815–1.870 times higher than that to the Midwest region to increase the likelihood of crowdfunding campaign success. Nevertheless, the monetary donation of crowdfunding campaigns helping people who live in the West region (*p* > 0.10) is insignificant. It implies that the effects of monetary donation to the Midwest and West regions on the success of crowdfunding campaigns are indistinguishable. H4 is supported

Regarding H5, in Models 3, 4, 5, and 6, picture is a positive and significant (*p* < 0.05) predictor of the likelihood of the success of crowdfunding campaigns. The odds ratio demonstrates that for each one-unit increase on picture, the odds of the success of crowdfunding campaigns increased by a factor of 1.537, 1.546, 1.580, and 1.642. Consequently, H5 is supported. Video is an insignificant (*p >* 0.1) variable in the Models 4, 5 and 6. Therefore, H6 is not supported. In terms of social network in Models 5 and 6, the social network is a positive and significant (*p* < 0.05) predictor of the likelihood of the success of the crowdfunding campaigns. The odd ratios (1.428 and 1.433) show that when increasing one unit of a social network, the increased odds of the success of crowdfunding campaigns are 1.428 and 1.433 times individually. H7 is supported. For the variable, external website, in model 6, it is insignificant (*p* > 0.1), indicating no effect on the success of a crowdfunding campaign. Hence, H8 is not supported. In all models, update is a positive and significant predictor of the likelihood of the success of crowdfunding campaigns. The odds ratio indicates that for each one-unit increase on Update, the odds of the successful crowdfunding campaigns increased by a factor of 1.530, 1.535, 1.524, 1.526, 1.564, and 1.621. H9 is supported.

Similarly, comment is also a positive and significant predictor (*p* < 0.05), which representing while increasing one unit of comment, the odds of successful crowdfunding campaigns will increase 1.675, 1.675, 1.674, 1.671, 1.782, and 1.823 times. H10 is supported as well. In the light of follower, it is dummy coded using the 0–250 followers as the reference group. Compared with the 0–250 followers, crowdfunding campaigns with ≥1001 followers (*p* < 0.05) are 1.540–1.566 times, 501–1000 followers (*p* < 0.05) are 1.284–1.334 times, and 251–500 followers (*p* < 0.10) are 1.216–1.265 times higher than 0–250 followers to cause the likelihood of the success of crowdfunding campaigns. H11 is supported. Lastly, for testing H12, 0–500 shares are set as a reference group. The results show that crowdfunding campaigns with ≥ 1501 shares (*p* < 0.05) are 1.701–1.714 times, 1001–1500 followers (*p* < 0.05) are 1.543–1.548 times, and 501–1000 followers (*p* < 0.10) are 1.271–1.284 times higher than 0–500 followers to cause the likelihood of the success of crowdfunding campaigns. H12 is supported as shown in Table 6.

## 5. Discussion and Conclusions

Previous studies about crowdfunding campaigns before the COVID-19 pandemic mainly focused on (1) the success factors of offline social causes campaigns in relation to where, whom, how, and when [19], (2) whether project’s goal and subject, geography, duration, number of backers and amount funded are successful determinants [73], (3) discussing crowdfunding performance with campaigns, crowd funders, crowdfunding platforms, and fund-seeker-related factors [74], (4) investigating the effects of backer endorsement and creator credibility on the success to crowdfunding campaigns [9], (5) exploring pledgers’ motivations such as funding duration, target amount, and word, video, and image counts [75], (6) the relationship between project funding and duration, and fundraising success [76], and (7) identifying the key drivers of a successful crowdfunding campaign through a machine learning approach [77]. As Briggs et al. [78] mentioned that during COVID-19, the general public firmly kept a solid attachment to the pre-lockdown normality, but this “event” had changed and stimulated a social change to a more fundamental change.

Therefore, our study focuses on how people react to crowdfunding campaigns after COVID-19 outbroke. In addition, for providing unique research contributions, this study not only explored the previous determinants of successful campaigns but also significantly extended to disentangle title word count (e.g., title word count was divided into 1–3, 4–5, 6–7, and ≥8-word groups), description word count (e.g., divided into 1–300, 301–600, 601–900, and ≥901-word groups), geographical locations (e.g., based on the most affected areas), number of followers (divided into 0–250, 251–500, 501–1000, and ≥ 1001 followers) and number of shares (divided into 0–500, 501–1000, 1001–1500, and ≥ 1501 shares) into small fractions for better understanding in which proper procedure that a campaign may accurately invoke people to participate in and increase the success probability of crowdfunding campaigns. Crowdfunding campaigns provide opportunities for fundraisers to look for potential donors. The study measures what determinants could lead to the success of a crowdfunding campaign in an empirical test that has not been explored previously.

The title of a crowdfunding campaign is a significantly condensed summary. The findings show that comparing with different title characters, the title of a crowdfunding campaign with four to five words plays an essential role in the success of a crowdfunding campaign. This setting may get donors’ attention and understand the title idea easily. The fundraiser needs to make sure that only a few words contain the critical essential information to backers [79]. Too many title words may lead to donors’ low motivation to read the title; in the country, one to three words may cause the idea of a crowdfunding campaign not easy to be understood. This finding is consistent with the tip of GoFundMe [41] that short title words have a high success probability of a crowdfunding campaign.

As expected, increasing the word count of descriptions in a crowdfunding campaign may transfer enough information about the campaign to donors. This study reveals that 600–900 words in the description section are associated with the highest success probability in a crowdfunding campaign. Crowdfunding donors are interested in a descriptive text to justify the reasons for crowdfunding as it could be considered a valuable signal in conveying precise message contents. While previous studies mainly demonstrated a positive linear relationship between word count and the successful probability of a crowdfunding campaign [17,46], this study presented if the word count in the description exceeds over 901 words, the campaign is still effective to persuade donors to donate money, but the effect is more minor than 600–900 words. Compared with a description including >901 words, using 600–900 words to illustrate the purpose of a crowdfunding campaign increases the successful probability by 29.1%. Too many words may result in low patience to understand the campaign. In contrast with too many words, too few words may let potential donors worry about the risks and increase the uncertainties [36] because donors hope to alleviate their concerns about information asymmetry and the campaign fundraiser’s credibility. Hence, preparing appropriate 600–900 words in the description of a campaign would be optimal and beneficial for donors to get involved with the campaign.

The result shows that a negative correlation exists between spelling errors and the successful probability of a crowdfunding campaign. It is consistent with the research of Mollick [23], which specifies that spelling errors may reduce the chance of crowdfunding success. Furthermore, spelling error implies that fundraisers are careless and thoughtless, and they do not prepare the campaign thoroughly. It also creates an untrustworthy impression on donors.

The location test was measured to find whether the crowdfunding campaigns’ success varied rested on the region with high infected numbers or low infected numbers. The results show that crowdfunding campaigns will have a high success probability when money is going to help people who live in the Northeast (odd rates: 1.873 and 562,629 infected cases) and South (odd rates: 1.858 and 445,087 infected cases) regions where the infected numbers among four regions in the United States continent have the top two infected cases. The odd rates in the Northeast region are higher than 0.8% in the South region. The findings are aligned with the research of Mollick [23], indicating that crowdfunding donation is more concentrated to give people who are in need.

The crowdfunding campaign with pictures presented provides additional information associated with the fundraiser or organization behind the campaign and enhances the reality of the campaign. The visual presentation is a more influential storytelling instrument than word-only description [15]. The results show that the presence of any picture positively affects the successful probability of a crowdfunding campaign. It is in line with previous studies [36,57], which point out that while the fundraisers put more effort into preparing a visual pitch, it will cause a positively successful rate of a crowdfunding campaign. In addition, the visual preparation could provide more detailed and direct information and let potential donors understand and get more involved with the campaign easily. However, the presence of a video is insignificant, which supports previous studies [26,46,60], stating that crowdfunding campaigns do not benefit from the existence of video presentations. Lagazio and Querci [46] stated that a video introducing the crowdfunding campaigns irrespective of the existence of a text description reduces funding chances by 5%.

Concerning social networks, our results demonstrate that the presence of any social network link such as Facebook or YouTube will cause a successful probability of a crowdfunding campaign. The results suggest that a fundraiser of a crowdfunding campaign should consider sharing and circulating the campaign to the general public. It may also lead to higher online searchability and affect donors’ decision-making [15]. In this way, it will cause a successful crowdfunding campaign, supporting previous findings [55,58].

In terms of an external website link, this study demonstrates that if a crowdfunding campaign fundraiser provides an external website link to the general public, it will not evoke potential donors’ motivation to understand the campaign, which supports the study of [60,80]. The result is inconsistent with previous studies [17,32,46] that point out the functionality of an external website link to not only dimmish the level of information asymmetry but also increase the probability of success. Maybe one of the reasons is that the donors during the pandemic are much more concerned with social connectedness with fundraisers during a natural disaster through social media, updates, and comments on how they can cope with traumatic life events [81].

Regarding the presence of any update in a crowdfunding campaign, it illustrates the degree to fundraisers’ close attention on the campaign. The findings show that a positive relationship between the update existence and campaign success, which supports the studies of [46,60] that updates imply a positive quality signal, solving asymmetric information, increasing the attraction, and reducing potential donors’ confusion about a crowdfunding campaign. Keeping updated the crowdfunding campaign page is the best chance to deliver the message of new progress to the potential donors and motivate them to involve the campaign. Furthermore, updates are also an excellent opportunity to signify the enthusiastic attitude of the fundraiser.

The comment section serves as a communication channel between fundraisers and potential donors. Our findings present that the presence of any comment will positively impact the successful probability of a crowdfunding campaign. The results confirm the studies of [55,63] that the engaging interaction between fundraisers and potential donors is a symbol of success and will achieve and positive pledge ratio. Comments directly convey attitudes, perceptions, or attitudes of potential donors. As Bi et al. [82] point out, comments may make potential donors assume a physical effect on word of mouth and receive valuable signals from the campaign. As a consequence, comments are critical to a successful crowdfunding campaign.

The main functions of the follower and share on the crowdfunding platform are that follower shows how many potential donors’ concern about the campaign and share demonstrates an expectation to let more and more people recognize and know this campaign. The results show that crowdfunding campaigns with more followers and shares positively cause a higher probability of success. Especially in terms of followers, the success probability in the number of followers ≥1001 is 17% higher than it of 501–1000 and 24% higher than it of 251–500. Regarding the share, the number of shares ≥ 1501 is 10% higher than it of 1001–1500 and 33% higher than it of 501–1000. Our finding supports the research of [44]. When more followers and shares are associated with a crowdfunding campaign, it may attract more potential donors’ attention, decrease the information asymmetries [15] and influence the funding amount [66]. Therefore, comments and shares play essential roles to matter the success of a crowdfunding campaign as well. This study’s new finding contributes to sustainability issues in humanitarian effort and crowdfunding literature.

## 6. Implications

### 6.1. Theoretical Implications

The literature on DbC during a natural disaster or health crisis is still in its infancy. Our study complements existing research on DbC’s success determinants before the pandemic crisis. As the number of DbC studies increase [9,10,18,19,20,21], it is vital to have better conceptual models through which scholars can interpret and provide definite success factors. This study proposed a model and explained some contextual similarities and differences that negate the generic factors for successful crowdfunding campaigns to create better DbC campaigns strategies to induce potential donors to donate, especially during a health crisis.

This study provides several theoretical implications contributing to the knowledge of DbC fundraising campaigns on crowdfunding platforms. Among the few studies that have examined successful crowdfunding campaign strategies, primary attention has been given to equity-based and reward-based crowdfunding campaigns. As crowdfunding platforms are different by nature (e.g., equity, reward, lending, and donation-based platforms), Kaartemo [74] suggested that clear contextual success factors for a specific platform should be highlighted while discussing the generalization of previous research on the success of crowdfunding campaigns. Therefore, we identified the following research gaps. First, many scholars examined the determinants of crowdfunding success for equity-based crowdfunding [14,18,56,83] and reward-based crowdfunding [23,55], but little attention has been given to DbC in both conceptual work and empirical evidence. Second, there is a lack of underlying theories and theoretical models in the current DbC literature [9,84]. In addition, the majority of studies on the critical antecedents of success focus on equity-based and reward-based crowdfunding campaigns, leaving the DbC study underdeveloped [20].

Third, most preliminary literature applied qualitative research studies [2,10]. Further, and naturally, many interesting questions remain unanswered even though a number of people are using crowdfunding platforms to draw awareness to their illness stories and medical costs. This knowledge gap is filled in this research paper and responds to the successive calls for more studies on DbC. Fourth, this study highlights how fundraisers raise food relief in crowdfunding platforms during challenging circumstances like the coronavirus epidemic. It is likely to be different from previous research that focuses on the following: corporate social responsibilities [84], illnesses and disabilities campaigns [2], Ramadan-related projects [9], and various forms of DbC from pets, education, volunteers, and others [18] except food relief.

Fifth, while prior studies only focused on analyzing how signals influence the likelihood of obtaining financial resources and reduce information asymmetry [15,29,31], this study contributes to expanding the signaling theory of Ross [27] and Spence [28] and its effectiveness. Scholars have long recognized the challenges of information asymmetry in securing donations and proposed various strategies to mitigate this problem. This study builds on this stream of prior research on signal interactions. We extend previous research by systematically examining how signals originating from donation-based campaigns influence the likelihood of obtaining donations. We define and use three different signal success measures: (1) signals originating from the campaign, (2) signals originating from the fundraiser, and (3) signals originating from the social interaction of the fundraiser with the crowd. Thus, the results of this research provide significant analyses of the underlying antecedents of success in the presence of DbC. It enhances the theoretical by testing all elements we found in the signaling theory from previous studies.

### 6.2. Practical Implications

The practical implications of this study are valuable from different perspectives. First, the crisis in the healthcare and social system during the coronavirus pandemic shows that DbC campaigns provide a living struggle representation of individuals and families to cope with the current and long-term negative consequences of this pandemic. Further, it compels individuals to seek social and financial supports from various interactive social networks. Therefore, fundraisers should learn various marketing strategies on how to promote their DbC for financial survival. Despite DbC’s expanding significance, only 13% to 20% of overall DbC campaigns are successfully funded [7,85]. Still, many campaigns fail to yield favorable outcomes because of unsuccessful attempts to convince donors to donate [35].

Individuals, not-for-profit organizations, and non-governmental organizations (NGOs) who want to be funded for a cause by the mass public compete with a vast number of other DbC campaigns for attention and funding. Given that donors on DbC campaigns provide a voluntary act of kindness where donors do not expect any return, fundraisers should mitigate information asymmetry because the donors can be informationally disadvantaged [32,33]. The potential donors need time and effort to process the given information and credibility of the fundraiser and the campaign.

Therefore, from a practical perspective, fundraisers, in setting up their DbC campaign, fundraisers should continuously put ongoing attention and implement a proactive approach to their campaigns in order to ensure potential and existing donors’ engagement with them throughout the entire process of the project, especially in the absence of monetary and non-monetary benefits to the part of the donors.

Second, understanding various signals of success of DbC campaigns is valuable for individuals and families to have a strategic marketing mindset when initiating fundraising campaigns to attract more donations from the crowd. Further, the results of this study will be helpful to fundraisers in their effort to set up marketing campaigns using different elements of signaling theory. Third, it is important to fill the research gap, especially the coronavirus epidemic, which has significantly affected the whole world. It is urgent to have an in-depth analysis of this issue to develop a coronavirus DbC campaign more effectively.

Fourth, in the global pandemic context, the problem of food shortage has surged throughout the United States [86]. Meanwhile, people seek various sources to fulfill the intense demands of securing food safety to keep a normal life during global hardship. Among the sources, crowdfunding has been a prevailed medium to require food relief. At the same time, this research demonstrated that if people want to raise a successful crowdfunding campaign for food relief, they must immediately take immediate care of the content presented on the fundraising page. Further, setting a reasonable fundraising goal as donors’ economic concerns could be sensitive to this global hardship. Furthermore, trying to provide as much supplementary information as possible, such as the comments, updates, and social media information, could facilitate the decision-making by donors as this could demonstrate a compelling desire of the fundraisers for their demands.

Lastly, the research gap about the antecedent of success of DbC campaigns during the coronavirus epidemic is also well fulfilled. This research serves as the pioneer to identify the influencing features to the outcome of the campaign that no previous studies have addressed. Therefore, the study also adds value to the crowdfunding studies as there is a compelling result indicating that fundraising projects in different contexts (i.e., COVID-19) have various significant influencing factors to the campaign success.

### 6.3. Limitations and Future Research

While our findings are essential for understanding how technological advances, donation-based crowdfunding can assist individuals and families during challenging times. This research paper is subject to limitations that need future research to be followed up. Even though this study has been conducted with in-depth rigor, further research needs to be developed well. First, further research is required to refine the conceptual model and empirical tests related to DbC campaign success because this research data used only food relief campaigns from Gofundme.com during the coronavirus epidemic in the United States. Data from other countries and other crowdfunding studies could enhance the model of this research. The cross-cultural analysis of different signals that could influence donors from different countries is also an exciting research topic.

Second, besides the various signals identified in this paper, other variables can also influence potential and current donors to donate. Further research could explore signaling theory in various DbC campaigns such as illness, medical bills, unemployment, and funeral or memorial assistance during this epidemic challenges. Lastly, there are long-term consequences of the coronavirus epidemic. Individuals and families may need ongoing resource assistance in the months and even years following a pandemic. Therefore, there may be subsequent donation-based crowdfunding platforms created to assist people in need that our study cannot capture. These limitations point to a direction for future research.

## Figures and Tables

**Table 1:** Data characteristics of crowdfunding campaigns (*n* = 652).

All Selected Campaigns	Value
Fundraising goal, total	$12,975,214
Donation received, total	$6,348,256
Donors, total	62,495
Shares, total	158,952 times
Followers, total	63,587 people
Fundraising goal, median	$6658
Donation received, median	$1925
Number of donors, median	36
Nature of campaigns	
Buy food	573 (87.8%)
Buy and make food	21 (3.2%)
Make food	58 (9.0%)
Number of Covid-19 infected cases	
West region	215,532
Midwest region	367,675
South	446,108
Northeast	564,235

Note: Data period from 1 March–31 July 2020.

**Table 2:** The variables and descriptions.

Variables	Descriptions
Dependent Variable	
Crowdfunding Success	Whether the crowdfunding campaign reaches its target
Signals originating from the Campaign	
1. Title	Number of word(s)of the title of the crowdfunding campaign
2. Description	Number of word(s) on the description of the crowdfunding campaign
3. Spelling Error	Spelling error(s) in the description of the crowdfunding campaign
4. Location	Crowdfunding campaign donates to Midwest, Northeast, West, or South region. The standard of classification of the four regions is based on the criterion of the U.S. Census Bureau [68]
5. Picture	Photo(s) in the description of the crowdfunding campaign
6. Video	Video(s) to demonstrate the crowdfunding campaign
Signature originating from the fundraiser	
7. Social Network	Social network link(s) of the crowdfunding campaign
8. External Website	External website link(s) of the crowdfunding campaign
9. Update	Update(s) of the crowdfunding campaign
Signals originating from the social interaction of fundraiser with the crowd	
10. Comment	Comment(s) of the crowdfunding campaign
11. Follower	Number of follower(s) of the crowdfunding campaign
12. Share	Number of share(s) of the crowdfunding campaign

**Table 3:** Summary statistics of crowdfunding campaigns (*n* = 652).

Variables	Categories	Min	Max	Mean	SD	Frequency		Percentage	
						Yes	No	Yes	No
Dependent Variable									
1. Success	Yes: 1/No: 0	0	1	0.231	0.455	195	457	29.9	70.1
Signals originating from the campaign									
1. Title (words)	a. 1–3	2	3	2.520	0.499	19		2.9	
	b. 4–5	4	5	4.513	0.500	323		49.5	
	c. 6–7	6	7	6.535	0.501	256		39.3	
	d. ≥8	8	10	8.142	0.502	60		8.3	
2. Description (words)	a. 1–300	128	297	218.521	45.762	94		14.4	
	b. 301–600	304	598	450.860	77.028	123		18.9	
	c. 601–900	602	898	750.492	86.372	394		60.4	
	d. ≥901	904	1735	1315.275	260.535	47		6.3	
3. Spelling Error	Yes: 1/No: 0	0	1	0.641	0.491	418	234	64.1	35.9
4. Location	a. Midwest (1)	1	4	2.535	1.214	112		17.2	
	b. Northeast (2)					254		39.0	
	c. South (3)					202		31.0	
	d. West (4)					90		12.9	
5. Picture	Yes: 1/No: 0	0	1	0.694	0.445	442	210	67.8	32.2
6. Video	Yes: 1/No: 0	0	1	0.502	0.497	331	321	50.8	49.2
Signals originating from the fundraiser									
7. Social Network	Yes: 1/No: 0	0	1	0.658	0470	433	219	66.4	33.6
8. External Website	Yes: 1/No: 0	0	1	0.501	0.501	324	328	49.7	50.3
9. Update	Yes: 1/No: 0	0	1	0.375	0.478	251	401	38.5	61.5
Signals originating from the social interaction of Fundraiser with the crowd									
10. Comment	Yes: 1/No: 0	0	1	0.465	0.487	305	347	46.8	53.2
11. Follower (people)	a. 0–250	2	248	197.695	29.304	213		32.7	
	b. 251–500	253	498	366.7287	72.195	282		43.3	
	c. 501–1000	502	994	735.634	141.235	127		19.5	
	d. ≥1001	1002	8014	4289.854	1719.478	36		4.6	
12. Share (times)	a. 0–500	6	498	335.489	93.985	143		21.9	
	b. 501–1000	504	995	744,895	144.587	194		29.8	
	c.1001–1500	1004	1489	1241.236	138.729	251		38.5	
	d. ≥1501	1503	10,975	9002.328	4331.210	70		9.8	

**Table 4:** Correlation analysis.

Variables	1	2	3	4	5	6	7	8	9	10	11	12	13
1. Success	1												
2. SE	−0.108 **	1											
3. Picture	0.242 **	0.055	1										
4. Video	0.146	0.367	−0.048	1									
5. SN	0.351 **	0.129 **	−0.216	0.443 **	1								
6. EW	0.205	0.236	0.120 **	−0.177	0.212	1							
7. Update	0.145 **	0.183 *	0.311 **	0.198 **	0.162 **	0.257 **	1						
8. Comment	0.152 **	0.154 **	0.156 **	0.234	0.114 **	0.187 **	0.273 **	1					
9. Follower	0.134 **	−0.221 **	0.252	0.095 *	0.297 *	0.144 **	0.128 **	0.132 **	1				
10. Share	0.213 **	0.237	0.145 *	0.185 **	0.131 **	0.095 *	0.154 **	0.138 **	0.343 **	1			
11. Title	0.158 **	−0.222	0.162 **	0.290 *	0.065	0.085 *	0.276 **	0.206	0.204	0.228	1		
12. Des	0.168 **	0.203 **	−0.199 *	0.223 **	0.215 **	−0.128	−0.206	0.295 **	−0.127	−0.121 **	0.227	1	
13. Location	0.131 **	0.322	0.119 **	0.337	0.202	0.224 *	0.227 **	0.375	0.230 *	0.1212	0.104 **	−0.111	1

Note: ** *p*-value < 0.05; * *p*-value < 0.10. SE = spelling error, SN = social network, EW = external website, Des = description.

**Table 5:** Results of logistic regression analysis.

Variables	Model 1	Model 2	Model 3	Model 4	Model 5	Model 6
Signals Originating from the Campaign						
1. Title						
a. 1–3 (ref.)						
b. 4–5	1.762 ** (0.256)	1.762 ** (0.256)	1.758 ** (0.258)	1.757 ** (0.258)	1.797 ** (0.261)	1.812 ** (0.262)
c. 6–7	1.457 (0.258)	1.458 (0.259)	1.468 (0.261)	1.468 (0.261)	1.484 (0.264)	1.492 (0.264)
d. ≥8	1.091 (0.268)	1.092 (0.268)	1.094 (0.268)	1.095 (0.268)	1.055 (0.273)	1.068 (0.2732)
2. Description						
a. 1–300 (ref.)						
b. 301–600	1.160 (0.257)	1.162 (0.271)	1.159 (0.258)	1.159 (0.258)	1.153 (0.260)	1.152 (0.260)
c. 601–900	1.271 ** (0.239)	1.273 ** (0.24142)	1.271 ** (0.260)	1.266 ** (0.236)	1.271 ** (0.262)	1.263 ** (0.262)
d. ≥901	0.983 * (0.267)	0.985 * (0.270)	0.993 * (0.270)	0.993 * (0.270)	0.962 * (0.273)	0.965 * (0.272)
3. Spelling Error		0.656** (0.191)	0.660 ** (0.191)	0.660 ** (0.191)	0.669 ** (0.192)	0.672 ** (0.192)
4. Location						
a. Midwest (ref.)						
b. Northeast	1.864 ** (0.275)	1.867 ** (0.275)	1.865 ** (0.276)	1.869 ** (0.277)	1.894 ** (0.279)	1.879 ** (0.279)
c. South	1.870 ** (0.265)	1.872 ** (0.266)	1.873 ** (0.266)	1.874 ** (0.267)	1.844 ** (0.271)	1.815 ** (0.271)
d. West	1.478 (0.271)	1.481 (0.271)	1.492 (0.272)	1.492 (0.274)	1.423 (0.274)	1.403 (0.275)
5. Picture			1.537** (0.190)	1.546 ** (0.193)	1.580 ** (0.195)	1.642 ** (0.201)
6. Video				1.253 (0.182)	1.241 (0.184)	1.251 (0.184)
Signals Originating from the Fundraiser						
1. Social Network					1.428 * (0.208)	1.433 * (0.208)
2. External Website						1.021 (0.113)
3. Update	1.530 ** (0.191)	1.535 ** (0.190)	1.524 ** (0.191)	1.526 ** (0.196)	1.564 ** (0.196)	1.621 * (0.202)
Signals Originating from the Social Interaction of Fundraiser with the Crowd						
1. Comment	1.675 ** (0.186)	1.675 ** (0.187)	1.674 ** (0.187)	1.671 ** (0.187)	1.782 ** (0.190)	1.823 ** (0.191)
2. Follower						
a. 0–250 (ref.)						
b. 251–500	1.258 * (0.271)	1.257 * (0.272)	1.262 * (0.272)	1.265 * (0.272)	1.231 * (0.272)	1.216 * (0.275)
c. 501–1000	1.321 ** (0.259)	1.322 ** (0.260)	1.334 ** (0.262)	1.331 ** (0.262)	1.288 ** (0.265)	1.284 ** (0.263)
d. ≥1001	1.540 ** (0.261)	1.541 ** (0.261)	1.566 ** (0.261)	1.561 ** (0.261)	1.546 ** (0.263)	1.525 ** (0.191)
3. Share						
a. 0–500 (ref.)						
b. 501–1000	1.270 ** (0.239)	1.272 ** (0.240)	1.271 ** (0.271)	1.274 ** (0.265)	1.284 ** (0.274)	1.280 ** (0.275)
c. 1001–1500	1.543 ** (0.263)	1.543 ** (0.264)	1.544 ** (0.265)	1.548 ** (0.265)	1.546 ** (0.267)	1.546 ** (0.269)
d. ≥1501	1.708 ** (0.259)	1.709 ** (0.260)	1.711 ** (0.260)	1.714 ** (0.261)	1.709 ** (0.263)	1.701 ** (0.263)
Model fit indices						
*R*^2^ (Nagelkerke)	0.288	0.295	0.306	0.312	0.337	0.345
χ2	53.761 ***	53.782 ***	53.795 ***	53.817 ***	64.377 ***	65.598 ***
*df*	17	18	19	20	21	22

Note: odd ratios are presented; standard error in parentheses; * *p*-value < 0.10; ** *p*-value < 0.05; *** *p*-value.

**Table 6:** Summary of hypothesis testing results.

Hypotheses	Results
H1: More characters on the title → Crowdfunding Success	Partially Supported
H2: More word count in the description section → Crowdfunding Success	Partially Supported
H3: Presence of any spelling error → Crowdfunding Success	Supported
H4: Location with more COVID-19 infected rates → Receive more Crowdfunding Donations	Supported
H5: Presence of any picture → Crowdfunding Success	Supported
H6: Presence of any video → Crowdfunding Success	Not Supported
H7: Presence of any social network link → Crowdfunding Success	Supported
H8: Presence of any external website link → Crowdfunding Success	Not Supported
H9: Presence of any update → Crowdfunding Success	Supported
H10: Presence of any comment → Crowdfunding Success	Supported
H11: More followers → Crowdfunding Success	Supported
H12: More shares → Crowdfunding Success	Supported

## Data Availability

The data that support the findings of this study are available from the corresponding author, upon reasonable request.
